# Association of dog ownership with accelerometer-measured physical activity and daily steps in 70-year-old individuals: a population-based cross-sectional study

**DOI:** 10.1186/s12889-021-12401-4

**Published:** 2021-12-21

**Authors:** Marcel Ballin, Oskar Antonsson, Viktor Rosenqvist, Peter Nordström, Anna Nordström

**Affiliations:** 1 Department of Community Medicine and Rehabilitation, Unit of Geriatric Medicine, Umeå University, 90187 Umeå, Sweden; 2 Department of Public Health and Clinical Medicine, Section of Sustainable Health, Umeå University, Umeå, Sweden; 3 School of Sport Sciences, UiT the Arctic University of Norway, Tromsø, Norway

**Keywords:** Physical activity, Walking, Health promotion, Public health, Epidemiology

## Abstract

**Background:**

Dog ownership (DO) has been associated with higher levels of self-reported walking and physical activity. However, compared to device-based measures, self-reported measures of physical activity may suffer from bias due to recall and social desirability. They are also incapable of quantifying light-intensity physical activity (LPA) and step volume, both of which may have important health benefits, especially for older adults. In this study, we investigated the association of DO with accelerometer-measured physical activity of different intensities and daily steps in 70-year-old individuals.

**Methods:**

This was a population-based cross-sectional study including 1406 participants aged 70 years [54.1% female] who participated in a health survey in Umeå, Sweden between February 2017–November 2019. All participants self-reported DO [yes/no]. Daily averages of LPA, moderate-to-vigorous-intensity physical activity (MVPA), and steps per day [steps/d] were measured for 1 week using hip-mounted Actigraph GT3X+ accelerometers. Associations were investigated using linear- and logistic regression models, adjusted for sociodemographic and health-related factors, date of examination, and accelerometer wear time.

**Results:**

The prevalence of DO was 14.1% [*N* = 199]. After adjustment for all covariates, DO was associated with 19.2 more minutes/d of LPA [95% CI, 8.8–29.6], 11.4 more minutes/d of MVPA [95% CI, 8.0–14.9] and 1738 more steps/d [95% CI, 1326–2149]. DO was also associated with twice the odds of meeting the physical activity recommendations [OR, 2.07, 95% CI, 1.48–2.90]. Exploratory interaction analyses showed that the association between DO and steps/d was stronger [P_interaction_ = 0.030] in female [β = 2165, 95% CI, 1585–2744] than in male [β =1255, 95% CI, 664–1845], with a similar trend for MVPA [P_interaction_ = 0.082].

**Conclusions:**

In this study of community-dwelling 70-year-old individuals, DO was associated with higher levels of daily LPA, MVPA, and steps. With the limitation of the observational design of the study, these findings add knowledge regarding the beneficial role that DO may play for promoting physical activity in the older population. In turn, these findings could support the development and evaluation of targeted interventions seeking to promote dog-friendly environments and facilitate dog walking in the community.

**Supplementary Information:**

The online version contains supplementary material available at 10.1186/s12889-021-12401-4.

## Background

Physical inactivity is a strong risk factor for non-communicable diseases and premature mortality [1], and has been associated with substantial economic burden [2]. More than one third of the population in developed countries has been estimated to be physically inactive [3], and the prevalence increases with age [4]. Because the world’s population is ageing rapidly [5], targeting physical inactivity in older people is of particular importance. Given the age-related decline in levels of moderate-to-vigorous-intensity physical activity (MVPA), recent evidence suggesting that light-intensity physical activity (LPA) is associated with health benefits [6–9] is of particular interest. Similarly, favorable associations have been observed also between daily steps and health outcomes [10, 11], which is interesting given that walking is the most preferred type of physical activity [12], as well as because step count is an intuitive metric of total physical activity. Therefore, identifying factors associated with physical activity of any intensity in older adults would be important.

Encouraging dog walking has been proposed as a strategy for population-level promotion of physical activity [13], especially in developed countries where the prevalence of dog ownership (DO) is high. Roughly 40% of households in the US [14] and Australia [15] own a dog, and 15% of households in Sweden [16]. Even though reviews have highlighted an association between DO and higher levels of walking and physical activity [13, 17], most studies have so far estimated walking and physical activity through measures of self-report [13, 17], which may suffer from bias due to recall and social desirability. This leads to an overestimation of physical activity. In contrast, device-based measures provide more accurate estimates and are better for assessment of incidental physical activity such as LPA, as well as step count [18, 19]. However, the current evidence on DO and device-measured physical activity of different intensities is limited. One study found that DO was associated with higher levels of device-measured LPA but not MVPA [20], although the study included only middle-aged individuals. Another study found that DO was associated with 2760 more steps per day [steps/d] in older adults, but only 86 participants were included [21]. Finally, a larger study found that DO was associated with up to 22% higher levels of device-measured total physical activity in older adults [22]. However, specific intensities were not investigated, and available covariates were few and self-reported [22]. For example, although adjustments were made for self-reported health and education, there were no data on marital status, income, or objectively measured health such as preexisting disease, body weight or physical function. These factors may influence either DO, physical activity, or both [23–25].

Collectively, the association between DO and device-measured physical activity of different intensities in older adults has been insufficiently investigated. Apart from a small number of studies, limitations include lack of studies conducted in population-based samples, lack of investigation of multiple intensities of physical activity, and lack of adjustment for objectively assessed covariates which could influence DO and physical activity. In this cross-sectional study, we investigated the associations of DO with accelerometer-measured LPA, MVPA and steps/d in a population-based sample of 70-year-old women and men, while adjusting for a variety of both subjectively and objectively assessed sociodemographic and health-related factors.

## Methods

### Study design and population

This was a population-based, cross-sectional study, conducted in Umeå, a municipality in northern Sweden with 130,224 residents in December 2020. The study was based on the ongoing, population-based, primary prevention study Healthy Ageing Initiative (HAI), which aims to identify risk factors for major non-communicable disease. The HAI started in 2012 and invites all 70-year-old individuals living in Umeå to an extensive health examination. The health examination consists of a comprehensive test battery, where a research nurse leads the participant through an extensive number of measurements and assessments, including both objective and subjective parameters of health. The eligibility criteria for participation in HAI is being 70 years of age and living in Umeå municipality. Since the start of the study, about 70% of the total population of 70-year-old individuals in Umeå have participated [26]. Of all eligible participants whose contact information has been available and who subsequently responded to the invitation, about 84% have agreed and participated in the study [26].

In HAI, assessment of DO was incorporated into the test battery in February 2017. Thus, individuals eligible for inclusion in the present study were all participants in HAI between February 2017 and November 2019 who also had complete data on DO, physical activity, and all covariates as described below. Thus, there was no missing data for any participant in the present study.

### Ethical considerations

Ethics approval for the present study was obtained from the Regional Research Ethical Review Board of Umeå University, Sweden (no. 07-031 M with extensions). All participants provided written informed consent to participate and were made aware of their possibility to voluntarily terminate their participation at any time. The study was conducted in accordance with the World Medical Association’s Declaration of Helsinki.

### Assessment of dog ownership

DO was assessed by the research nurse asking the participant if they own or take care of any dog [yes/no]. No further questions were asked regarding for example the breed of the dog or the time spent walking the dog.

### Assessment of physical activity

Physical activity was assessed using hip-mounted Actigraph GT3X+ accelerometers. The participants were told to wear the device for 1 week and to remove it when showering or bathing. All HAI participants until April 2018 were instructed to remove the device also when sleeping. From May 2018 and onwards, the protocol was changed so that participants were instructed to wear the accelerometer during nighttime if feasible. The raw data were collected at 30 Hz frequency and filtered using the standard Actigraph filter to eliminate non-human accelerations. Using Actilife 6.11.3 software, the raw data were transformed into “counts” of movements during 1-min intervals, also known as the epoch length. Count data were classified as LPA or MVPA using the Freedson cut-points [27], and steps were assessed using the proprietary Actigraph algorithm. Periods of non-wear time were defined using the Choi et al. algorithm [28]. To be included, participants were required to have > 10 h of daily wear time for > 4 days. The total amount of LPA, MVPA, and steps during the week of registration was divided by the number of valid wear days for each participant to calculate daily averages. In addition, adherence to physical activity recommendations were defined as those accumulating on average > 30 min/d of MVPA. This definition has been used in previous studies in the same population [8, 26], and the rationale for defining adherence to the recommendations in this way was that not all participants wore the accelerometer for the entire week. Thus, the recommendations were translated into daily averages in order to account for differences in the number of valid wear days among participants.

### Covariates

In HAI, body weight and height were measured using a digital scale [HL 120; Avery Berkel, Fairmont, MN, USA] and a gauge [Holtain Limited; Crymych, Dyfed, UK]. The body mass index (BMI, kg/m^2^) was calculated by dividing body weight by height squared. Physical function was assessed using the Timed-Up-and-Go (TUG) test [29]. Participants self-reported current smoking status [yes/no]. Depressive symptoms were assessed using the Geriatric Depression Scale 15-item version [GDS-15] [30].

From Statistics Sweden [the national agency for statistics, www.scb.se], we obtained individual-level data on marital status [widowed/divorced/never married/married], annual disposable household income, and highest level of education [primary/secondar/post-secondary], obtained from the age of 65 for each participant. From the National Inpatient Register and the National Outpatient Register, managed by the National Board of Health and Welfare [www.socialstyrelsen.se], we obtained data on history of cardiovascular disease using *International Classification of Diseases, 10th Revision* (ICD-10) codes. The National Inpatient Register includes all diagnoses set in inpatient care since 1987, and the National Outpatient Register covers all secondary outpatient care since 2001. Reporting to these registers is mandatory by law. Using these registers, we tracked previous diagnoses of myocardial infarction [I21], angina pectoris [I20], stroke [I61-I64], and heart failure [I50].

### Data linkage

The HAI data was linked with the registry data through the following steps. First, the HAI data was sent to Statistics Sweden who attached data on socioeconomic status and replaced the Personal Identification Number with a unique pseudonymized identifier for each participant. Next, Statistics Sweden forwarded the data and the code list to the National Board of Health and Welfare who attached data on cardiovascular disease, before returning all the files to us. Finally, were merged all individual files together to form a combined file for data analysis.

### Statistical analysis

Descriptive data were presented as means with standard deviations or as frequencies with percentages. Differences between dog-owners and non-dog owners were tested for using t-test for independent samples (continuous variables) or chi-square test (categorical variables). For the primary analysis, linear regression models were used to estimate unstandardized beta values (β) with 95% confidence intervals (CI) for the association between DO and each of the physical activity variables modelled on a continuous scale [minutes/d of LPA, minutes/d of MVPA, and number of steps/d]. In a secondary analysis, binary logistic regression was used to estimate odds ratios (OR) with 95% CI for the association between DO and meeting physical activity recommendations [yes, > 30 min/d MVPA, no, < 30 min/d MVPA]. All models were first performed unadjusted. Next, a fully-adjusted model was performed, including the covariates sex [categorical variable], accelerometer wear time [continuous variable], date of examination [month as a categorical variable], BMI [continuous variable], TUG [continuous variable], smoking status [categorical variable], GDS-15 score [continuous variable], cardiovascular disease [categorical variable], level of education [categorical variable], marital status [categorical variable], and annual disposable household income [continuous variable]. The fully-adjusted models for the analyses of DO with LPA and MVPA were mutually adjusted for each other. Multicollinearity was explored using tolerance and variance inflation factor. The tolerance and variance of inflation factor values were between 0.8–0.9 and 1.0–1.1 for all covariates, hence there was no evidence of multicollinearity [31].

To test whether the associations differed by sex, BMI, or time of year for examination, explorative interaction analyses were performed by creating product terms between DO and each of these variables, which were added to the fully-adjusted model. These analyses were exploratory and were conducted based on evidence indicating that these factors may potentially influence dog walking [22, 24].

Finally, a sensitivity analysis was conducted to investigate whether the results were influenced by the change in accelerometer wear protocol which occurred during the study, where participants from May 2018 and onwards wore the accelerometer during nighttime. Thus, we repeated the analyses after excluding all participants from May 2018 and onwards.

All analyses were performed using Stata MP version 16.1 for Mac [StataCorp, College Station, TX 77845, USA]. Statistical significance was determined as either *P* < 0.05 or as 95% CIs for the β values which did not cross 0, or as 95% CIs for the ORs which did not cross 1.

## Results

### Participant characteristics

**Fig. 1:**
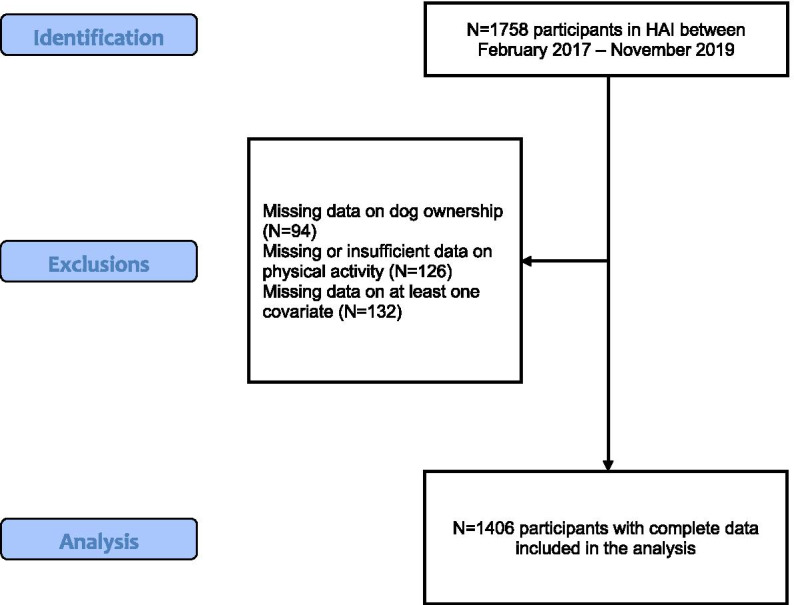
Study flow chart

A flow chart of participant eligibility, exclusion, and inclusion is presented in Fig. 1. In short, a total of 1758 individuals participated in HAI between February 2017 and November 2019, of which 1664 had data on DO. From these 1664 individuals, 1538 individuals (92.4%) had valid data also on physical activity. After excluding participants with missing data for any of the covariates, the final study population comprised 1406 individuals who were included in the analysis. The mean age of participants was 70.3 years and 54.1% were female. A total of 199 participants reported DO [14.1%]. Detailed participant characteristics for the total sample and according to DO status is shown in Table 1.

**Table 1:** Participant characteristics for the total study sample and by dog ownership status

Variables	Total (***N*** = 1406)	Dog owners (***N*** = 199)	Non-dog owners (***N*** = 1207)	***P*** for difference between groups
Age, yrs, mean (SD)	70.3 (0.2)	70.3 (0.2)	70.3 (0.2)	0.6
Female sex, n (%)	761 (54.1)	101 (50.8)	660 (54.7)	0.3
Current smoker, n (%)	52 (3.7)	4 (2.0)	48 (4.0)	0.2
**Month of examination, n (%)**				0.2
January	58 (4.1)	14 (7.0)	44 (3.7)	
February	72 (5.1)	11 (5.5)	61 (5.1)	
March	125 (8.9)	21 (10.6)	104 (8.6)	
April	159 (11.3)	17 (8.5)	142 (11.8)	
May	178 (12.7)	28 (14.1)	150 (12.4)	
June	131 (9.3)	15 (7.5)	116 (9.6)	
July	1 (<0.1)	1 (0.5)	0	
August	165 (11.7)	25 (12.6)	140 (11.6)	
September	145 (10.3)	20 (10.1)	125 (10.4)	
October	176 (12.5)	20 (10.1)	156 (12.9)	
November	140 (10.0)	20 (10.1)	120 (9.9)	
December	56 (4.0)	7 (3.5)	49 (4.1)	
**Health parameters**				
BMI, kg/m^2^, mean (SD)	26.5 (4.1)	26.8 (4.4)	26.4 (4.1)	0.3
Timed-up-and-go test, seconds, mean (SD)	9.5 (2.1)	9.5 (2.0)	9.5 (2.1)	0.9
GDS-15 score, mean (SD)	1.2 (1.8)	1.4 (1.7)	1.2 (1.8)	0.2
History of cardiovascular disease, n (%)	174 (12.4)	34 (17.1)	140 (11.6)	0.029
**Socioeconomic data**^**a**^				
Annual disposable household income, 1000 SEK, mean (SD)	487.7 (295.8)	469.7 (256.4)	490.7 (301.8)	0.4
**Education, n (%)**				1.0
Primary	146 (10.4)	21 (10.6)	125 (10.4)	
Secondary	557 (39.6)	80 (40.2)	477 (39.5)	
Post-secondary	703 (50.0)	98 (49.3)	605 (50.1)	
**Marital status, n (%)**				0.5
Widowed	52 (3.7)	10 (5.0)	42 (3.5)	
Divorced	215 (15.3)	35 (17.6)	180 (14.9)	
Never married	150 (10.7)	20 (10.1)	130 (10.7)	
Married	989 (70.3)	134 (67.3)	855 (70.8)	
**Accelerometer measurements, mean (SD)**				
LPA, mins/day	269.0 (76.2)	283.2 (77.4)	266.7 (75.8)	0.0045
MVPA, mins/day	34.1 (24.5)	43.4 (30.3)	32.6 (23.1)	<0.001
Steps/day	7355 (3104)	8712 (3724)	7131 (2932)	<0.001
Valid wear days	7.3 (0.7)	7.3 (0.7)	7.3 (0.7)	0.5
Wear time, hours/day	18.2 (4.1)	18.2 (4.0)	18.2 (4.1)	0.8
Meeting physical activity recommendations, n (%)*	690 (49.1)	122 (61.3)	568 (47.1)	<0.001

*Abbreviations*: *BMI* Body mass index, *GDS-15* Geriatric Depression Scale 15-item version, *IQR* interquartile range, *LPA* Light-intensity physical activity, *MVPA* Moderate-to-vigorous intensity physical activity, *SD* standard deviation, *SEK* Swedish Krona

^a^ Obtained from the age of 65 for all participants

^b^ Defined as accumulating on average >30 min/d of MVPA

### Dog ownership and physical activity

The results from the regression models are shown in Table 2. In unadjusted models, DO was significantly associated with higher levels of physical activity of all intensities, and with higher probability of meeting physical activity recommendations. The strength of the associations increased after adjustment for all covariates. Specifically, DO was associated with 19.2 more minutes/d of LPA [95% CI, 8.8–29.6], 11.4 more minutes/d of MVPA [95% CI, 8.0–14.9], and with 1738 more steps/d [95% CI, 1326–2149]. Expressed in relative terms, this corresponded to 7.2% more minutes/d of LPA/d, 35.0% more minutes/d of MVPA, and 24.4% more steps/d for dog owners compared to non-dog owners. Furthermore, DO was associated with twofold higher odds of meeting physical activity recommendations [OR, 2.07, 95% CI, 1.48–2.90].

**Table 2:** Associations between dog ownership and physical activity in the total cohort of 1406 participants

Dog owners compared to non-dog owners	Increase in steps/day β (95% CI)	Increase in LPA/day β (95% CI)	Increase in MVPA/day β (95% CI)	Meeting physical activity recommendations OR (95% CI)^a^
Unadjusted	1581 (1122–2039)	16.6 (5.2–28.0)	10.8 (7.2–14.5)	1.78 (1.31–2.42)
Adjusted^b^	1738 (1326–2149)	19.2 (8.8–29.6)	11.4 (8.0–14.9)	2.07 (1.48–2.90)

^a^Defined as accumulating on average > 30 min/d of MVPA

^b^All adjusted models included the following covariates: sex, date of examination, BMI, physical function, smoking status, GDS-15 score, cardiovascular disease, level of education, marital status, annual household disposable income, accelerometer wear time. The analyses of LPA and MVPA were mutually adjusted for each other

*Abbreviations*: *β* unstandardized beta, *BMI* body mass index, *CI* confidence interval, *GDS-15* Geriatric Depression Scale 15-item version, *LPA* light-intensity physical activity, *MVPA* moderate-to-vigorous intensity physical activity, *OR* odds ratio

#### Interaction analyses

The association between DO and steps/d was stronger [P_interaction_ = 0.030] in female [β = 2165, 95% CI, 1585–2744] than in male [β =1255, 95% CI, 664–1845]. Similarly, there was a non-significant trend suggesting a stronger association [P_interaction_ = 0.082)] between DO and minutes/d of MVPA in female [β = 14.4, 95% CI, 9.8–19.0] than in male [β = 8.0, 95% CI, 2.7–13.2]. In contrast, sex did not modify the association between DO and LPA [P_interaction_ = 0.5]. Finally, neither BMI nor time of year of examination modified the association between DO and either LPA, MVPA, or steps [P_interaction_ > 0.2 for all interactions].

#### Sensitivity analysis

For the sensitivity analysis, a total of 775 participants who wore the accelerometer during nighttime were excluded, leaving a total of 631 participants for inclusion in the analysis. Of these, 89 participants [14.1%] were dog owners, similar as in the main analysis. The results of the regression models are presented in Supplemental Table 1. This sensitivity analysis confirmed the findings from the main analysis.

## Discussion

In this population-based study of community-dwelling 70-year-old individuals, DO was associated with higher levels of daily LPA, MVPA, and steps, and with higher odds of meeting physical activity recommendations. Exploratory interaction analyses suggested that the associations of DO with steps and MVPA was stronger among female participants, but none of the associations differed based on BMI or time of year for examination.

One of the main results of this study was the favorable association of DO with LPA. We found that dog owners performed 19 more min/d of LPA compared to those not owning a dog. To our knowledge, there has been no previous study of the association between DO and LPA in older adults. However, our findings extend those from a study on DO and accelerometer-measured physical activity in Japanese middle-aged individuals, showing a similar estimate [20]. Emerging evidence suggest that LPA may potentially have implications for health in older adults. In a cohort study, an increase in LPA by 30 min/d was associated with 13% lower risk all-cause mortality in older men [32]. Other studies in older adults have observed beneficial associations between LPA and subjective well-being [33, 34], metabolic syndrome [26], mobility disability [35], and cardiovascular disease [8, 9].

Another key result was that dog owners took roughly 1700 more steps/d compared to those not owning a dog. This is about 1000 steps/d lower than what was found in a previous longitudinal case-controlled study in older adults [21]. However, that study was likely not conducted in a representative sample, because it included a very small number of volunteers. Our study sample may better represent a general population. When the estimates in the present study were expressed in relative terms, dog owners took 24.4% more steps/d compared to those not owning a dog. This finding is supported by the results from a population-based study from the UK, which showed that DO was associated with up to 22% higher levels of accelerometer-measured total physical activity in older adults [22]. Moreover, we found that the association between DO and steps/d was stronger in females compared to in males. We are not aware of any similar results reported in the literature, and a previous systematic review found mixed evidence regarding whether sex correlates with dog walking [24]. Therefore, we may only speculate about potential explanations for this result. One explanation could be perceived feelings of neighborhood safety, which has been shown to correlate with dog walking among females [24]. However, it should be noted that our interaction analyses were exploratory, hence these findings are preliminary and warrants further investigation. Regardless, there is evidence to suggest that such an increment in daily steps observed in both male and female may potentially be clinically relevant. For instance, cohort studies including both older men and women showed that every 500 steps/d and every 1000 steps/d was associated with about 10% lower risk of cardiovascular disease [36] and about 15% lower risk of mortality [32, 37]. Another cohort study found beneficial associations also with incident falls [38]. Findings related to step volume may also have practical implications because it represents an intuitive metric that is easy to understand for the general population, and because steps can be easily monitored using devices that have become increasingly common.

Interestingly, we also found that dog owners accumulated 11 more minutes/d of MVPA. In a previous study from Japan, DO was associated with about 7 more minutes/d of accelerometer-measured MVPA in middle-aged adults [20]. However, that estimate was not statistically significant, perhaps in part due to the smaller study sample. Another possible explanation for the different result in that study compared to our study could be demographic factors. The study from Japan included individuals in working age who have more limited time for leisure time physical activity such as MVPA. In contrast, our study population comprised older individuals above retirement age who would have more time to recreationally walk their dog. Another factor that could contribute to the beneficial association in the present study may have to do with environmental factors, which have been shown to correlate with dog walking [24]. The municipality of Umeå, wherein the present study was conducted, is characterized by a dog-supportive physical environment, including walking areas in proximity and high access to public spaces and a variety of green- and outdoor areas. Importantly, measures of the physical environment have been proposed as key [24] when it comes to promoting dog walking in the community and in turn increase population levels of physical activity. Altogether, the favorable associations found in the present study between DO and higher levels of physical of all intensities therefore suggest that efforts to promote dog walking in the community may help increase population levels of physical activity in older adults. However, given the lack of similar studies conducted in older adults, further population-based studies are warranted, including also older adults of a wider age span and from other regions.

This study has some important strengths, including the relatively large population-based sample with a high participation rate (84% of all invited) and a high adherence to wearing the accelerometer (92.4%). Together, this reduces selection bias and increases the possibility to generalize the findings to other cohorts of community-dwelling 70-year-old individuals. We were also able to adjust our analyses for several objectively assessed covariates, either from clinical examinations or though national registries with nationwide coverage, which minimizes information bias. Also, the assessment of physical activity using accelerometers is a notable strength. In a previous study based on the same population, self-reported physical activity was largely overestimated as compared with accelerometer-measured physical activity [39].

Some limitations should also be noted. First, the cross-sectional design prevents causal inferences from being drawn, although there is some prospective data supporting a link between DO, dog acquisition and increased physical activity [40, 41]. Moreover, although we adjusted our analyses for several covariates, there is the potential of residual and unmeasured confounding. For example, data on dog characteristics was lacking, which may be thought to potentially influence the associations. However, a systematic review found mixed evidence for an association between dog characteristics such as dog breed, and dog walking [24]. Also, because socioeconomic data were obtained from age 65 for all participants, t socioeconomic factors may have changed between age 65 and study participation at age 70. For example, if some participants retired during this period this would affect annual income. However, we deem the risk of bias due to changes in annual income to be low given that official data show that about 80% of people in Sweden born during 1947–1949 (the same year as participants in the present study) had retired by the age of 65 [42]. Another factor to consider is that the accelerometer wear protocol was changed during the study, which may have introduced bias. However, a sensitivity analysis excluding participants wearing the accelerometer during nighttime confirmed the results of the primary analysis. Next, the accelerometer cut-points used to classify physical activity intensities were based on absolute intensity and originally validated in younger people [27]. In older adults, this could potentially result in misclassification between LPA and MVPA. However, by using these cut-points we have previously shown associations of LPA with cardiovascular disease and mortality [8]. Also, given the relatively high mean physical activity in this population as compared with other populations of similar age [33, 43], these cut-points appear to be plausible for use in the present population. Regarding step count, the accelerometer used in the present study may slightly underestimate the total number of steps, yet it correlates strongly with other commercial step count devices [44].

## Conclusions

To summarize, this cross-sectional study conducted in a population-based sample of 70-year-old individuals showed that DO was associated with higher levels of daily LPA, MVPA, and steps. This adds knowledge regarding how DO may play a beneficial role in promoting physical activity in the older population. The findings could support the development and evaluation of targeted interventions seeking to promote dog-friendly environments and facilitate dog walking in the community.

## Supplementary Information

**Additional file 1.**

## Data Availability

The datasets analyzed during the current study are not publicly available due to regulations under Swedish law but are available from the corresponding author on reasonable request.

## Abbreviations

BMI: Body mass index
CI: Confidence interval
DO: Dog ownership
GDS-15: Geriatric Depression Scale 15-item version HAI Healthy Ageing Initiative
ICD-10: International Classification of Diseases, 10th Revision
OR: Odds ratio
LPA: Light-intensity physical activity
MVPA: Moderate-to-vigorous-intensity physical activity
TUG: Timed-Up-and-Go
