# Supplementary material for: Association of dog ownership with accelerometer-measured physical activity and daily steps in 70-year-old individuals: a population-based cross-sectional study
Source: BMC Public Health. 2021 Dec 21;21:2313. doi: 10.1186/s12889-021-12401-4 (PMC8691041; doi:10.1186/s12889-021-12401-4)
Supplement: Supplementary file 1 — Additional file 1. [file 12889_2021_12401_MOESM1_ESM.docx]

| **Supplemental Table 1.** Associations between dog ownership and physical activity in 631 participants included in the sensitivity analysis. | | | | |
| --- | --- | --- | --- | --- |
| **Dog owners compared to non-dog owners** | **Increase in steps/day β (95% CI)** | **Increase in LPA/day  β (95% CI)** | **Increase in MVPA/day β (95% CI)** | **Meeting physical activity recommendations OR (95% CI)^a^** |
| Unadjusted | 1321 (609-2033) | 14.1 (-2.4-30.7) | 8.6 (3.0-14.2) | 1.59 (1.01-2.50) |
| Adjusted^b^ | 1528 (886-2170) | 16.1 (0.9-31.3) | 9.9 (4.5-15.3) | 1.92 (1.16-3.17) |
| ^a^Defined as accumulating on average >30 min/d of MVPA.  ^b^All adjusted models included the following covariates: sex, date of examination, BMI, physical function, smoking status, GDS-15 score, cardiovascular disease, level of education, marital status, annual household disposable income, accelerometer wear time. The analyses of LPA and MVPA were mutually adjusted for each other.  **Abbreviations:** β **=** unstandardized beta, BMI = body mass index, CI = confidence interval, GDS-15 = geriatric depression scale 15-item version, LPA = light-intensity physical activity, MVPA = moderate-to-vigorous intensity physical activity, OR = odds ratio | | | | |
